# Genetic loci associated with winter survivorship in diverse lowland switchgrass populations

**DOI:** 10.1002/tpg2.20159

**Published:** 2021-10-18

**Authors:** Hari P. Poudel, Neal W. Tilhou, Millicent D. Sanciangco, Brieanne Vaillancourt, Shawn M. Kaeppler, C. Robin Buell, Michael D. Casler

**Affiliations:** ^1^ Agriculture and Agri‐Food Canada Lethbridge AB Canada; ^2^ Dep. of Agronomy Univ. of Wisconsin‐Madison Madison WI USA; ^3^ Dep. of Plant Biology Michigan State Univ. East Lansing MI USA; ^4^ USDA‐ARS U.S. Dairy Forage Research Center Madison WI USA

## Abstract

High winter mortality limits biomass yield of lowland switchgrass (*Panicum virgatum* L.) planted in the northern latitudes of North America. Breeding of cold tolerant switchgrass cultivars requires many years due to its perennial growth habit and the unpredictable winter selection pressure that is required to identify winter‐hardy individuals. Identification of causal genetic variants for winter survivorship would accelerate the improvement of switchgrass biomass production. The objective of this study was to identify allelic variation associated with winter survivorship in lowland switchgrass populations using bulk segregant analysis (BSA). Twenty‐nine lowland switchgrass populations were evaluated for winter survival at two locations in southern Wisconsin and 21 populations with differential winter survivorship were used for BSA. A maximum of 10% of the individuals (8–20) were bulked to create survivor and nonsurvivor DNA pools from each population and location. The DNA pools were evaluated using exome capture sequencing, and allele frequencies were used to conduct statistical tests. The BSA tests revealed nine quatitative trait loci (QTL) from tetraploid populations and seven QTL from octoploid populations. Many QTL were population‐specific, but some were identified in multiple populations that originated across a broad geographic landscape. Four QTL (at positions 88 Mb on chromosome 2N, 115 Mb on chromosome 5K, and 1 and 100 Mb on chromosome 9N) were potentially the most useful QTL. Markers associated with winter survivorship in this study can be used to accelerate breeding cycles of lowland switchgrass populations and should lead to improvements in adaptation within USDA hardiness zones 4 and 5.

## Core Ideas

- Bulk segregant analysis (BSA) is a low‐cost alternative to association studies for identifying underlying QTL.
- Lowland switchgrass planted in southern Wisconsin exhibited differential winter hardiness.
- BSA test revealed 9 QTL in tetraploid and 7 QTL in octoploid populations.
- QTL at 88 Mb on chromosome 2N, 115 Mb on 5K, 1 and 100 Mb on 9N are deemed potential.
- Fixation of allele for winter survivorship in lowland population was not observed.

**Abbreviations**
4x: tetraploid
8x: octoploid
BP: breeding populations
BSA: bulk segregant analysis
FDR: false discovery rate
GWAS: genome‐wide association studies
LD: linkage disequilibrium
*NDPK*
: *NUCLEOSIDE DIPHOSPHATE KINASE*
QTL: quantitative trait loci
*PHYB*
: p*hytochrome B*
SNP: single nucleotide polymorphism
TAV: trait‐associated variants.

## 1 INTRODUCTION

Switchgrass (*Panicum virgatum* L.) is a native North American perennial grass with potential as a biofuel feedstock due to its high biomass potential on marginal lands with limited inputs. There are two ecotypes of switchgrass, lowland and upland, both of which are relevant to the development of biofuel feedstocks, largely dependent on geographic region (Porter, 1966; Zalapa et al., 2011). The lowland ecotypes are adapted to more southerly climes, whereas upland ecotypes are found in more northern latitudes (Casler, 2005, 2012; Casler et al., 2007; Casler et al., 2004; Lovell et al., 2021). Lowland and upland ecotypes are phenotypically distinct as exhibited by differences in overall height, leaf blade dimensions, tiller number, and heading/flowering dates (Casler, 2005).

The lowland ecotype of switchgrass has generated considerable interest, largely due to its extremely late flowering characteristics (Grabowski et al., 2017; Tornqvist et al., 2018). In USDA hardiness zones 3–5, lowland switchgrass flowers 4–6 wk later than upland switchgrass, increasing the duration of the biomass accumulation phase and increasing biomass yields by up to 50% (Casler et al., 2004). Multiple‐location field evaluations of highly selected late‐flowering populations confirmed a 50% increase in biomass yield, with the added benefit of broadening the adaptation range of lowland switchgrass from hardiness zones 6–9 to include hardiness zones 4 and 5 (Casler et al., 2018). However, lowland germplasm derived from prairie and savanna remnants in the southern United States (USDA hardiness zones 6–9) have low winter survivorship at the northern latitudes (Casler, 2012; Casler & Vogel, 2014; Casler et al., 2004). Lowland cultivars planted in northern Wisconsin (USDA hardiness zone 3b) had up to fourfold yield and winter survival loss compared with Oklahoma (USDA hardiness zone 7a) (Casler et al., 2004). Lovell et al. (2021) reported 42% winterkill in lowland plants compared with only 2% in upland plants when planted in northernmost sites in the United States (SD, NE, IL, and MI). These authors also reported a strong correlation between extreme minimum temperature of the site of origin with the biomass yield of lowland populations. As yield and winter survival are highly correlated (Bhandari et al., 2011; Lovell et al., 2021), the sustainable and economic use of switchgrass as an energy grass in northern latitudes can be improved by increasing winter survivorship of lowland ecotypes.

Previous experiments have documented that most mortality of switchgrass occurs when the plants are in their winter dormant phase (Sarath et al., 2014). Cold tolerance, generally measured as survivorship following stressful winter conditions (cold temperatures, high winds, frequent freeze‐thaw cycles, and open winters with little or inconsistent snow cover), is a heritable trait (Hopkins et al., 1995) and can be improved by simple phenotypic selection methods (Casler et al., 2018; Poudel et al., 2020). However, this process requires many years to ensure adequate levels of stress and to accumulate sufficient changes in allele frequency to warrant the release of a new cultivar. Identification of DNA markers associated with winter hardiness in lowland switchgrass would be extremely valuable to support marker‐based selection programs and accelerate the assessment and selection of winter‐hardy lowland genotypes.

Many traits of agronomic importance in crop plants are quantitatively inherited, meaning that they are controlled by many quantitative trait loci (QTL) with small individual effects (Falconer & Mackay, 1996). Identification of these QTL would provide the basis for marker‐based selection approaches that reduce selection cycle time through its effectiveness on early‐stage selection and allow selection in the absence of selective forces. Mapping QTL in experimental populations (Morton, 1955) and genome‐wide association studies (GWAS) across diverse populations (Klein et al., 2005; Price et al., 2010; Zhu et al., 2008) are the most common approaches used for the identification of trait‐associated variants (TAV). Conventional QTL mapping is based on newly generated recombination events which are often population‐specific (Feltus et al., 2006). On the other hand, GWAS exploit historical recombination within a large diversity panel and provide higher resolution to the mapping of the quantitative traits (Price et al., 2010; Zhu et al., 2008). Both of these approaches require genotyping of a large number of individuals, which is labor‐intensive, time‐consuming, and costly. Researchers working on cold tolerance in switchgrass are still in the initial stages of identification of TAV. To date, six QTL have been identified for freezing tolerance of switchgrass; however, these are likely specific to one particular lowland × upland hybrid population (Poudel et al., 2019b).

Bulk segregant analysis (BSA) is an alternative approach to identify TAV, which involves genotyping pooled DNA from individuals with shared phenotype that significantly reduce genotyping cost (Michelmore et al., 1991). This method relies on the differences of allelic frequencies between the phenotypically distinct pools and can be implemented with any type of marker data (Magwene et al., 2011). Traditionally, BSA studies were performed in biparental mapping studies. However, next‐generation sequencing‐based BSA approaches are also equally applicable in detecting QTL with small effects in diversity panels, given that effective population size and selection intensity are both adequate (Ehrenreich et al., 2010; Magwene et al., 2011). Hence, with the advent of high‐throughput genotyping technologies and the availability of a reference genome, BSA is becoming an increasingly useful method of identifying TAV in both biparental mapping populations and diverse populations.

In this study, we performed BSA to identify genetic loci related to winter survivorship in lowland switchgrass populations. The lowland populations were subjected to natural winter pressure at two locations in southern Wisconsin. We hypothesized that alleles favorable to cold hardiness would be enriched in individuals that survive winters and that these alleles would be underrepresented in individuals that fail to survive winters. Therefore, using the differential rate of survival following winter stress, DNA pools were constructed for each population by bulking samples into survivor and nonsurvivor pools. These DNA pools were genotyped via exome capture sequencing. Using allele frequencies, we identified markers associated with winter survivorship in lowland populations.

## 2 MATERIALS AND METHODS

### 2.1 Plant materials and experimental design

Twenty‐nine diverse lowland switchgrass populations from the United States and Mexico (Table 1), representing a wide range of geographic areas, were used to test winter survival in this study. Eighteen populations were collected as prairie remnants in autumn 2014 (natural populations) and the other 11 populations were derived from breeding populations (BP) previously described (Evans et al., 2018; Grabowski et al., 2017). These BP were all derived from lowland populations collected across the southern United States and previously subjected to selection for winter survivorship in USDA hardiness zones 4 and 5. These 11 populations were planted in Normal, IL, in 2012, allowed to open pollinate, and their seeds harvested in 2014.

**TABLE 1:** Information and survivorship of the 29 lowland switchgrass populations used for cold hardiness screening and the number of plants used to create bulk DNA samples

				Arlington, WI				Madison, WI			
Population	Origin state^a^	Population type^b^	Ploidy	Total plants	Dead plants	Living plants	DNA pool^c^	Total plants	Dead plants	Living plants	DNA pool^c^
WALS‐101	AL	NP	8x	82	72	10	8	74	38	36	8
WALS‐102	AL	NP	8x	188	132	56	19	193	73	120	20
WALS‐103	GA	NP	8x	188	162	26	19	189	108	81	19
WALS‐106	LA	NP	4x	184	182	2	NA	188	147	41	19
WALS‐109	MS	NP	4x	157	153	4	NA	165	127	38	17
WALS‐110	MS	NP	8x	148	90	58	15	140	77	63	14
WALS‐113	OK	NP	4x	173	98	75	18	184	13	171	13
ACC‐J249.BF.K	TX	NP	4x	188	159	29	19	193	84	109	20
ACC‐J250.BF.K	TX	NP	4x	187	167	20	19	192	110	82	20
ACC‐J251.BF.K	TX	NP	4x	170	162	8	8	179	98	81	18
SW‐20‐101	KS	NP	4x	171	153	18	18	156	69	87	16
SW‐28‐102	TX	NP	4x	184	146	38	19	186	107	79	19
SW‐28‐110	TX	NP	4x	186	162	24	19	192	47	145	20
ACC‐J268.BF.K	Mexico	NP	4x	188	185	3	NA	186	180	6	NA
SW‐28‐104	TX	NP	4x	182	144	38	NA	188	61	127	NA
SW‐28‐106	TX	NP	4x	186	153	33	NA	191	71	120	NA
SW‐28‐109	TX	NP	4x	185	150	35	NA	191	81	110	NA
SW‐28‐111	TX	NP	4x	164	125	39	NA	169	71	98	NA
Timber	NC, SC	BP	4x	147	65	82	15	151	29	122	16
SW788	NY	BP	4x	150	59	91	15	154	8	146	8
SW790	MS	BP	4x	186	134	52	19	192	46	146	20
Kanlow	OK	BP	4x	187	77	110	19	193	18	175	18
SWG39	NA	BP	4x	180	101	79	18	191	31	160	20
ECS‐6	MD	BP	4x	100	56	44	10	104	22	82	11
SW‐1641	KS	BP	4x	187	107	80	19	192	22	170	20
SW‐6‐2013	MS	BP	4x	187	175	12	12	176	97	79	18
SW789	AR, MS	BP	4x	186	105	81	NA	194	48	146	NA
SWG32	NA	BP	4x	182	112	70	NA	193	36	157	NA
SW‐2236	MS	BP	4x	78	30	48	NA	79	7	72	NA
Total				4,881	3,616	1,265		4,975	1,926	3,049	
Survivorship (%)					26				61		

^a^ NA, not available, because no specific state can be assigned.

^b^ Population type: NP, natural population, BP, breeding populations.

^c^ NA, not available because the population were not selected for bulk segregant analysis.

In February 2015, seeds of each population were germinated in plastic seedling tubes containing a 1:1 mixture of topsoil and peat moss in the glasshouse at the U.S. Dairy Forage Research Center, Madison, WI. The seeds were cold stratified for 15 d prior to seeding. Seedlings that were 16 wk of age were transplanted to one of two locations: Arlington, WI (Plano silt loam; fine‐silty, mixed, superactive mesic Typic Argiudoll), or Madison, WI (Kegonsa silt loam; fine‐silty over sandy or sandy‐skeletal, mixed, superactive, mesic Mollic Hapludalf), in June 2015. The experimental design was a randomized complete block design with eight blocks at each location. Plots consist of one row of up to 25 plants on a 0.3‐m spacing with 0.9 m between rows. Although Arlington and Madison testing sites are located in the same USDA hardiness zone 4b, these two locations were chosen because of differences in soil type and 30‐yr mean temperature. Weeds were controlled with pre‐emergence herbicides and hand weeding as described by Casler (2008). Plants were allowed to grow throughout the 2015 season and their biomass was removed after a killing frost.

There was little winter injury during the first winter, 2015–2016, with >95% survivorship for all populations at both locations. Both experiments were kept weed‐free in 2016 using herbicides and hand weeding as described above. Prior to senescence, leaf samples (approximately 5 cm) were collected from each plant in August 2016. Identical sized tissue samples were freeze‐dried, sealed into airtight plastic bags, and stored at room temperature until the phenotypic scoring of survivorship was recorded following the second winter, 2016–2017. A paper hole‐puncher was used to sample an equal amount of leaf tissue of all genotypes. Biomass of all plants was again removed after killing frost.

**Core Ideas**

- Bulk segregant analysis (BSA) is a low‐cost alternative to association studies for identifying underlying QTL.
- Lowland switchgrass planted in southern Wisconsin exhibited differential winter hardiness.
- BSA test revealed 9 QTL in tetraploid and 7 QTL in octoploid populations.
- QTL at 88 Mb on chromosome 2N, 115 Mb on 5K, 1 and 100 Mb on 9N are deemed potential.
- Fixation of allele for winter survivorship in lowland population was not observed.

Individual plants were scored twice for winter survival at approximately 10–30 d after the initial spring regrowth by visual assessment of the percentage of living shoots on the scale of 0–20, where 0 = dead, 1 = one green shoot, …, and 20 = no winter damage (Poudel et al., 2019a). The winter survivorship scoring system was intended to approximate a linear estimate of crown recovery, where a score of 20 was intended to translate to 100% of crown shoot survival. Based on the phenotypic scores for each population within a location, an equal number of plants was selected and bulked to create survivor and nonsurvivor tissue pools from each location, such that each pool contained no more than 10% (*n* = 8–20 individuals) of the original population size. The survivor pool consisted of samples with high survival scores and the nonsurvivor pool consisted of randomly selected dead plants (score = 0) while ensuring they represented all blocks within a location. Finally, 40 pairs of DNA pools (19 from Arlington and 21 from Madison) were constructed and used for DNA sequencing and further analysis based on the evidence of differential winterkill. For example, WALS_106 and WALS_109 did not show differential winterkill in the Arlington location and thus were not used in subsequent bulk segregant analysis (Table 1). The names of bulked samples consisted of a combination of evaluation location (A = Arlington and M = Madison), population name, and survivorship (0 = nonsurvival, 1 = survival).

### 2.2 Genotyping

Exome capture sequence data were generated using the NimbleGen SeqCap EZ Switchgrass Exome probe set as described previously (Tornqvist et al., 2018). Sample libraries were pooled equally and sequenced at the University of Wisconsin Biotechnology Center DNA Sequencing Facility on a HiSeq 2500 generating 101nt single‐end reads with a target sequencing coverage of 250×. Raw reads were cleaned using BBMap v37.61 (Bushnell, 2014), and read quality was assessed before and after trimming using FastQC v0.11.5 (http://www.bioinformatics.babraham.ac.uk/projects/fastqc/) and MultiQC v1.2 (Ewels et al., 2016). Cleaned reads for each sample were then aligned to the switchgrass reference genome (Pvirgatum‐450‐v4.0.hardmasked.fa) using BWA‐MEM v0.7.16a (Li, 2013) (http://bio‐bwa.sourceforge.net/bwa.shtml) and piped to SAMTools v1.5 (Li et al., 2009) for conversion to BAM format and sorting. BAM files from the same sample were then merged and sorted using SAMTools v1.5 (Li et al., 2009); duplicate reads were marked using Picard v2.7.2 (https://github.com/broadinstitute/picard/releases/tag/2.7.2). Local realignment was performed using the Realigner Target Creator and Indel Realigner tools from GATK v3.7.0 (McKenna et al., 2010) to minimize the number of mismatched bases across all reads. Sorted BAM files and pileup files were generated using the SAMTools v1.5 (Li et al., 2009) mpileup command with BAQ computation disabled and map quality adjustment disabled. Read data (allele counts) were extracted for 5,596,351 bi‐allelic loci previously identified from three switchgrass diversity panels (Acharya, 2014; Evans et al., 2014; Evans et al., 2018). These markers were then filtered for minor allele frequency <0.05 (356,741 markers remaining) and for mean read depth per sample between 15 and 600 (329,033 markers remaining) calculated across all populations. The later restriction was to avoid single nucleotide polymorphism (SNP) markers that might be inaccurately mapped to repetitive portions of the genome.

### 2.3 Statistical analysis

An association test based on an individual population could result in *p*‐value inflation due to unobserved population structure. Population structure is a concern because of prior reports that showed switchgrass can maintain mixtures of distant ancestral groups even in natural populations (Evans et al., 2018; Morris et al., 2011). To minimize false‐positive associations, the analysis targeted markers that were significant in multiple populations and in both evaluation environments. To cluster groups of populations with similar genetic backgrounds, the populations were clustered based on genetic similarity using *k*‐means cluster analysis in the R package stats. For each marker, a generalized linear mixed model was created predicting allele frequencies of all populations within a population group. Each model predicted allele frequencies as a binomial random variable with the read depth (the sum of reference and alternate allele reads) as the number of trials (i.e., logistic regression) (Yang et al., 2015). The binomial success probability was a linear function with the following fixed effects: survival status (survivor vs. nonsurvivor), population, and the interaction between population and survival status. The environment (Madison vs. Arlington) was included as a random variable. The test statistic for TAV significance was the likelihood ratio between models with and without survival status included. The ratio between models with and without the interactive effect between survival status and the population was also collected to estimate the reliability of the marker effect across multiple populations.

As discussed by Yang et al. (2015), the above statistic is prone to inflation and therefore two strategies were implemented to reduce inflation. First, a smoothing tri‐cubic kernel with a sliding window was used on the test statistic to reduce the likelihood of spurious relationships (Magwene et al., 2011). The appropriate smoothing window size was estimated through visual observations of test statistic distortion near strong peaks in the dataset. The smoothed test statistic was further corrected for inflation by using a regression approach genomic control strategy (λ [Devlin & Roeder, 1999] and R package gap). Briefly, the tri‐cubic smoothed test statistics was divided by the calculated inflation factor before calculating one‐tailed chi‐square distribution with one degree of freedom. Markers with false discovery rate (FDR) <0.05 were considered significant TAVs (Benjamini & Hochberg, 1995).

The post‐hoc analyses assessed the change in allele frequency of TAV within each population. The change in allele frequency between survival and nonsurvival pool (Δ) was calculated as

$$\Delta =\left(RC_{S}/\mathrm{TR}C_{S}\right)-\left(RC_{\mathrm{NS}}/\mathrm{TR}C_{\mathrm{NS}}\right)$$

where RC = read count of either the favorable or unfavorable allele, TRC = total read count, S = survivor pool (desirable allele), and NS = nonsurvivor pool (undesirable allele).

For each population and TAV, the two‐sided *p*‐values were calculated from Δ assuming the t‐distribution. The *p‐*values were then adjusted for FDR <0.01. In addition, the change in beneficial allele frequency among populations was assessed using linear regression on the minimum temperature of the coldest month (USDA hardiness zone) of the origin of the population. This test was conducted only in tetraploids. This test provided additional confidence to the identified QTL based on the knowledge that switchgrass exhibits latitudinal and longitudinal adaptability (Casler et al., 2007; Casler et al., 2004; Lovell et al., 2021; Poudel et al., 2019a). The annotation of the TAVs were accessed through Phytozome‐13 (https://phytozome‐next.jgi.doe.gov/ Goodstein et al., 2012).

## 3 RESULTS

### 3.1 Field‐based winter mortality

Following the second winter (2016–2017), there was differential mortality among populations at both locations (Table 1). The percentage of winter mortality ranged from 0 to 94% among populations. The lower mortality rate in 2015–2016 compared with 2016–2017 may be attributable to the higher minimum temperature during the early winter months (November and December), providing a longer period of cold‐acclimation (Guy, 1990). Even though the locations occurred in the same hardiness zone and both locations received similar low‐temperature stress (Supplemental Figure S1a), the survival in Madison was much higher than in Arlington. This difference could not be explained by either differential winter temperature or snow cover (Supplemental Figure S1b). Following the second winter, the mean percentage of living shoots was 9% at Arlington and 34% at Madison. Mean survivorship was 26% at Arlington and 61% at Madison (Table 1).

Natural populations tended to be lower in mean survivorship compared with the BP (mean difference = 19%, *p* < .01), perhaps because of their breeding and selection history. The highest winter survival for both locations was observed with population SW788, a breeding population originating from New York, whereas the lowest winter survival was observed in a population from Mexico, largely as expected based on geographic origins.

### 3.2 Sequencing read depth and population differentiation

**FIGURE 1:**
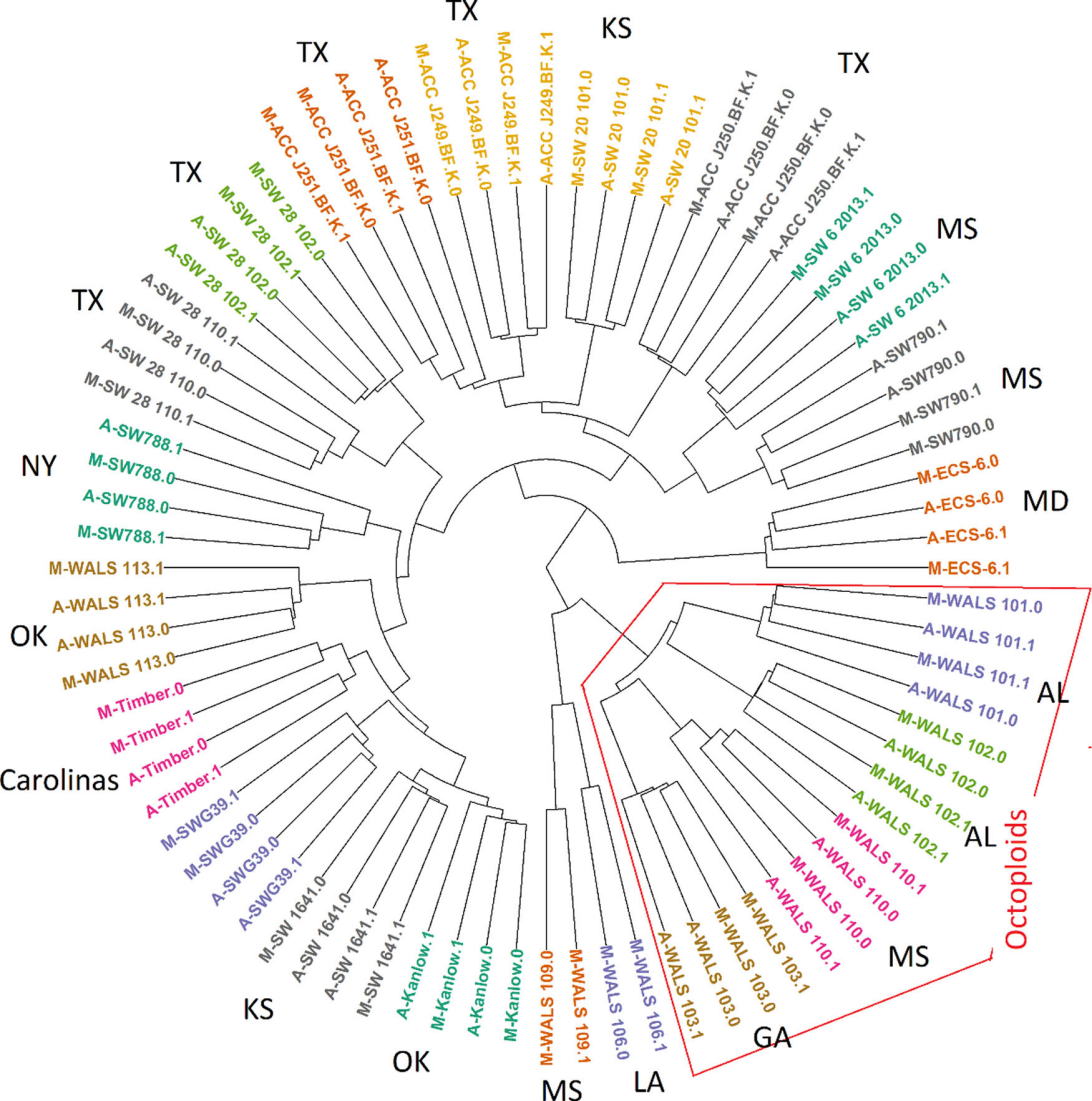
Dendrogram representing all 80 bulked DNA pools constructed using reference allele frequency from the filtered single nucleotide polymorphism (SNP) matrix (no. of SNP markers = 2.2 M). The labels of bulked samples consist of a combination of evaluation location (A = Arlington and M = Madison), population name and survivorship (0 = nonsurvival, 1 = survival). The labels printed outside the fan represent the origin of the populations. The state of origin of population SWG39 is unknown

The average sequencing read count across all samples was 64 (*N* = 329,033 SNPs), after removal of positions with extreme read counts (15 < read depth for all bulks < 600) and minor allele frequency (< 0.05). A dendrogram of the bulked DNA samples constructed using reference allele frequencies clearly differentiated the populations into two distinct clades and aligned with *k*‐means clustering (Figure 1). Four populations defined as octoploids (Poudel et al., 2019a) were monophyletic and separated from tetraploid populations, with the exception of two tetraploid populations: WALS_106 and WALS_109 from Louisiana and Mississippi, respectively. These two populations formed a distinct clade with a close resemblance to octoploids compared with tetraploids (Figure 1). The exact reason for these two populations having a higher resemblance to the octoploids is unknown; however, these two populations behaved differently during phenotypic evaluation, with distinctly highest winter mortality together with the population from Mexico in both years of evaluation. As such, those two populations were excluded from the association analysis. The average read depth in octoploids was greater than for tetraploids (Supplemental Figure S2a) in both the survivor and nonsurvivor pools (paired *t*‐test *p* < .001, mean difference = 4.5) similar to the results from Ramstein et al. (2018) in diverse switchgrass populations. Similarly, the standard error of sequencing depth was greater (paired *t*‐test *p* < .001) in octoploids in both survivor and nonsurvivor pools (Supplemental Figure S2b).

### 3.3 QTL discovery

**FIGURE 2:**
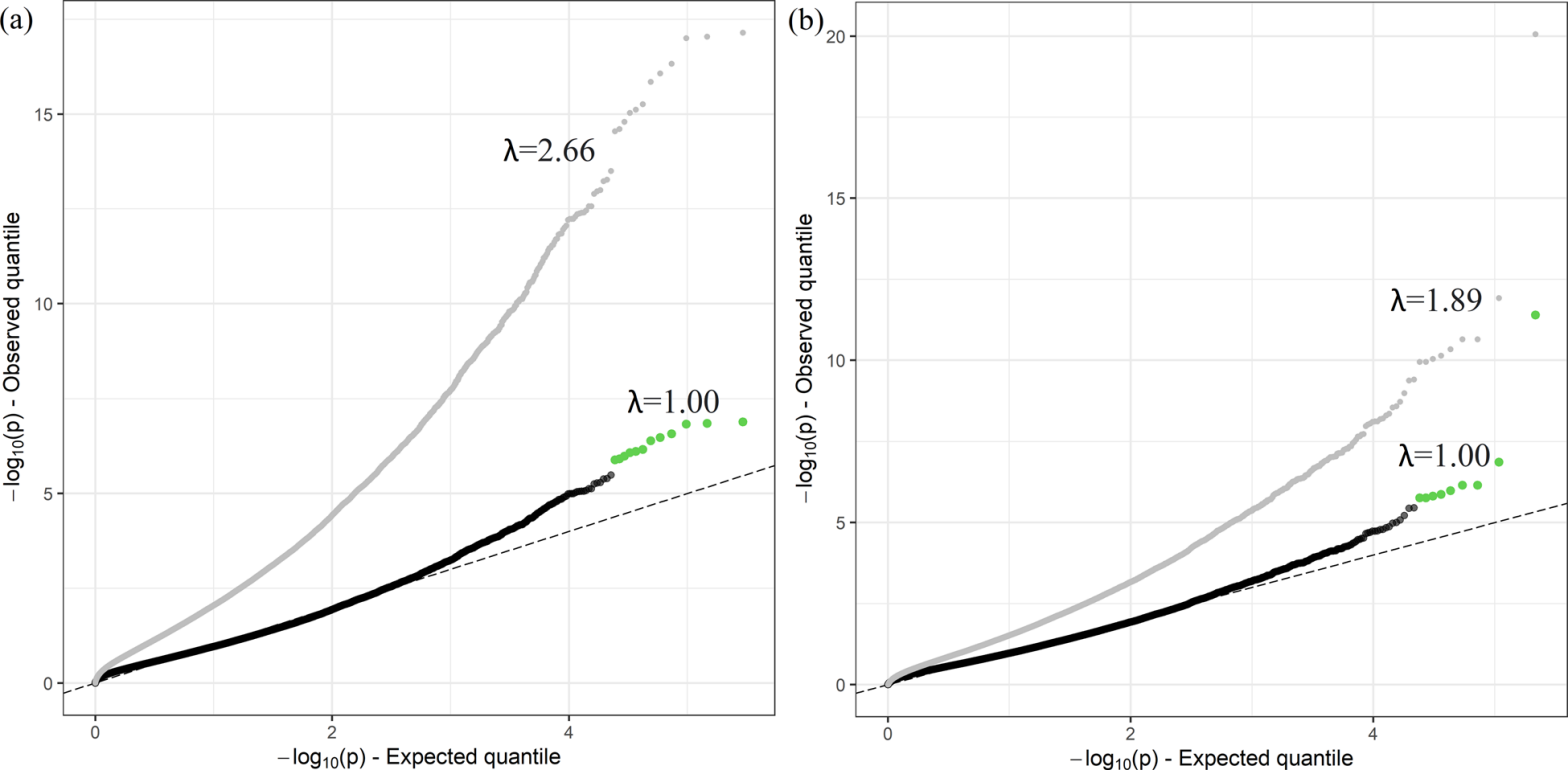
Q‐Q plot of the chi‐square estimates based on (a) 4x populations and (b) 8x populations. The gray dots and black dots represented the –log10(p) before and after control of genomic inflation factor respectively. The green dots are the single nucleotide polymorphisms (SNPs) significant at false discovery rate <0.05

Parallel association analyses were conducted using the bulked DNA samples from tetraploid populations (*n* = 60, with populations 106 and 109 excluded) and octoploid populations (*n* = 16) to eliminate any impacts of ploidy on the association analysis and provide a constant genetic background for TAVs. Visual evaluation of distortion near significance peaks indicated that test statistics revert to background levels well below 500 bp. The smoothing tri‐cubic kernel with a sliding window was therefore set to 250 bp. The genomic inflation factor (λ) of the smoothed function was 2.66 for the tetraploid populations and 1.89 for the octoploid populations (Figure 2a–b) prior to correction. Upon correction, the λ was approximately 1 for both tetraploids and octoploids (Figure 2a–b).

**FIGURE 3:**
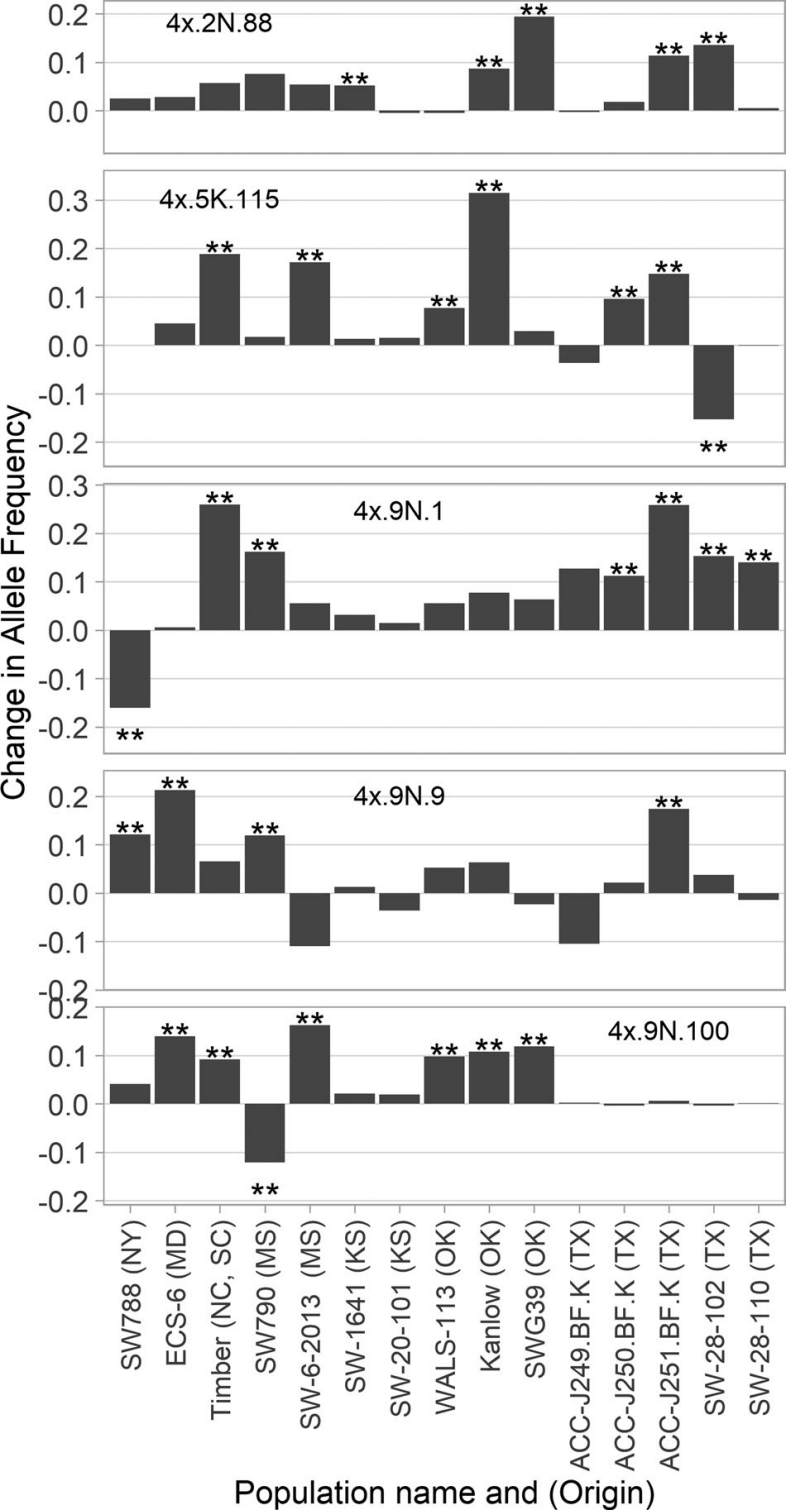
Change in allele frequency for five single nucleotide polymorphism (SNP) markers associated with selection for survivorship (difference between survivor and nonsurvivor groups) for 15 lowland switchgrass populations evaluated at two Wisconsin locations and classified according to state of origin (** indicates a significant selection effect at *p* < .01)

Using the tetraploid populations, 11 significant TAV comprising nine unique QTL were identified (Table 2). Note that the QTL in this paper are coded as ploidy (4x for tetraploid and 8x for octoploid), followed by chromosome number, and physical base position in Mb (e.g., 4x.9N.1 is a QTL identified using 4x populations at position 1 Mb on chromosome 9N). The post‐hoc *t*‐test analyses of these TAV indicated that the change in allele frequency between survival and nonsurvival pools (Δ) was different (*p* < .01) in at least three and at most seven out of 15 populations irrespective of the site of origin or type of population (Figure 3). Changes in allele frequencies between survival and nonsurvival pools of DNA for three QTL (4x.5K.115, 4x.9N.1, and 4x.9N.100) were different in up to seven populations. However, there was no evidence for fixation of alleles or selection signatures for winter survivorship between survivor and nonsurvivor pools in any of these QTL as indicated by the small differences in allele frequency (Figure 3, Supplemental Table S1). The greatest Δ, equivalent to 0.09, was observed in QTL 4x.9N.1and 4x.9N.9 (Table 2). Six of these 11 TAV corresponded to four unique QTL (4x.9N.1, 4x.9N.9, 4x.9N.26, and 4x.9N.100) located on chromosome 9N.

**TABLE 2:** List of quantitative trait loci (QTL) identified by using bulk‐segregant analysis in lowland switchgrass using the population model, including annotated gene description and functions

QTL‐ID^a^	SNP^b^	Δ^c^	Significant populations (*p* < .01)	Phytozome description
4x.2N.88	Chr02N_87628858	−0.06	Timber (NC, SC), Kanlow (OK), ECS‐6 (MD), ACC‐J250.BF.K (TX), ACC‐J251.BF.K (TX)	NUCLEOSIDE DIPHOSPHATE KINASE // SUBFAMILY NOT NAMED
4x.3K.35	Chr03K_34610981	−0.04	WALS‐113 (OK), Timber (NC, SC), SW790 (MS), Kanlow (OK), SW‐6‐2013 (MS), ACC‐J249.BF.K (TX)	COBRA‐like protein (COBRA)
4x.5K.115	Chr05K_115382984	0.05	WALS‐113 (OK), Timber (NC, SC), Kanlow (OK), SW‐6‐2013 (MS), ACC‐J250.BF.K (TX), ACC‐J251.BF.K (TX), SW‐28‐102 (TX)	No functional annotation
4x.5K.78	Chr05K_78372425	0.06	Timber (NC, SC), Kanlow (OK), ECS‐6 (MD), ACC‐J250.BF.K (TX), ACC‐J251.BF.K (TX)	No functional annotation
4x.7N.52	Chr07N_51529513	0.07	Timber (NC, SC), Kanlow (OK), ECS‐6 (MD), ACC‐J251.BF.K (TX)	shikimate O‐hydroxycinnamoyltransferase (E2.3.1.133, HCT)
4x.9N.1	Chr09N_546977	0.09	Timber (NC, SC), SW788 (NY), SW790 (MS), ACC‐J250.BF.K (TX), ACC J251.BF.K (TX), SW‐28‐102 (TX), SW‐28‐110 (TX)	solute carrier family 25 (mitochondrial S‐adenosylmethionine transporter),
4x.9N.9	Chr09N_8931909	0.04	SW788 (NY), SW790 (MS), ECS‐6 (MD), ACC‐J251.BF.K (TX)	histone H2A (H2A)
4x.9N.9	Chr09N_8931936	−0.09	Timber (NC, SC), SW788 (NY), ACC‐J251.BF.K (TX), SW‐28‐110 (TX)	histone H2A (H2A)
4x.9N.26	Chr09N_25765846	−0.06	WALS‐113 (OK), SW788 (NY), ACC‐J249.BF.K (TX), SW‐28‐102 (TX)	No functional annotation
4x.9N.26	Chr09N_25765898	−0.06	WALS‐113 (OK), SW‐20‐101 (KS), SW‐28‐102 (TX)	No functional annotation
4x.9N.100	Chr09N_100257480	0.04	WALS‐113 (OK), Timber (NC, SC), SW790 (MS), Kanlow (OK), SWG39 (OK), ECS‐6 (MD), SW‐6‐2013 (MS)	heat shock 70k Da protein 1/8 (HSPA1_8)
8x.1N.96	Chr01N_96391478	0.07	WALS‐103 (GA), WALS‐110 (MS)	AUXIN‐RESPONSIVE PROTEIN IAA10‐RELATED
8x.4K.62	Chr04K_61608303	−0.16	WALS‐101 (AL), WALS‐102 (AL), WALS‐110 (MS)	PPR repeat (PPR) // PPR repeat family (PPR_2)
8x.4K.9	Chr04K_8734544	−0.12	WALS‐101 (AL), WALS‐110 (MS)	ubiquitin‐conjugating enzyme E2 J2 (UBE2J2, NCUBE2, UBC6)
8x.4K.9	Chr04K_8734688	−0.15	WALS‐101 (AL), WALS‐110 (MS)	ubiquitin‐conjugating enzyme E2 J2 (UBE2J2, NCUBE2, UBC6)
8x.5K.114	Chr05K_113910680	−0.10	WALS‐101 (AL), WALS‐102 (AL)	CULLIN // SUBFAMILY NOT NAMED
8x.5K.114	Chr05K_113910681	−0.10	WALS‐101 (AL), WALS‐102 (AL)	CULLIN // SUBFAMILY NOT NAMED
8x.9K.8	Chr09K_7892756	0.04	WALS‐101 (AL)	5‐methyltetrahydropteroyltriglutamate–homocysteine methyltransferase (metE)
8x.9K.81	Chr09K_81213296	0.08	WALS‐101 (AL)	15‐cis‐phytoene desaturase (PDS, crtP)
8x.9N.49	Chr09N_49043534	0.13	WALS‐101 (AL), WALS‐102 (AL)	GINS complex subunit 4 (GINS4, SLD5)

*Note*. The phytozome description has been accessed through phytozome‐13 (https://phytozome‐next.jgi.doe.gov) (Goodstein et al., 2012).

^a^ QTL ID: 4x or 8x for ploidy plus chromosome identifiers and position of the QTL (in Mb) within each chromosome.

^b^ Physical position of the peak single nucleotide polymorphism (SNP) within a QTL based on *P. virgatum* reference genome version 4.1.

^c^ The difference in the allele frequencies between survival and nonsurvival pool averaged across all the populations.

**FIGURE 4:**
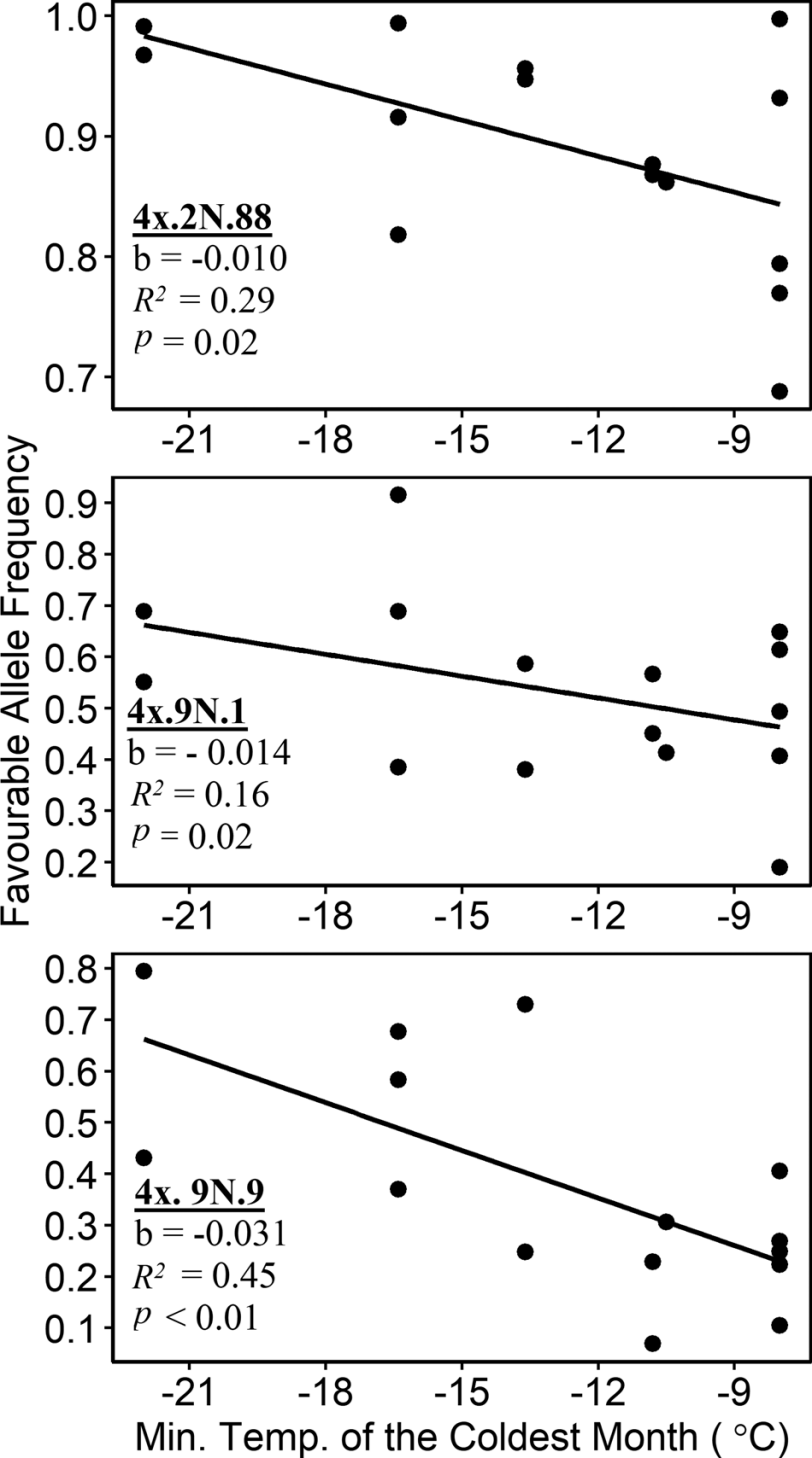
Linear regressions of favorable allele frequencies for three single nucleotide polymorphism (SNP) markers as a function of the mean temperature of the coldest month (30‐year mean) for 15 lowland switchgrass populations evaluated at two Wisconsin locations

The beneficial allele frequency decreased with the increase in minimum temperature of the coldest month of the site of origin of the population in only three out of nine QTL (4x.2N.88, 4x.9N.1, and 4x.9N.9) (Figure 4). This indicates that temperature was not a sole indicator of the change in allele frequencies. However, these three TAV provide additional support for the identified QTL. The association was greatest in QTL 4x.9N.9 (*p* < .01 and *R* ^2^ = 0.45) followed by QTL 4x.2N.88 (*p* = .02 and *R^2^* = 0.29) (Figure 4).

**FIGURE 5:**
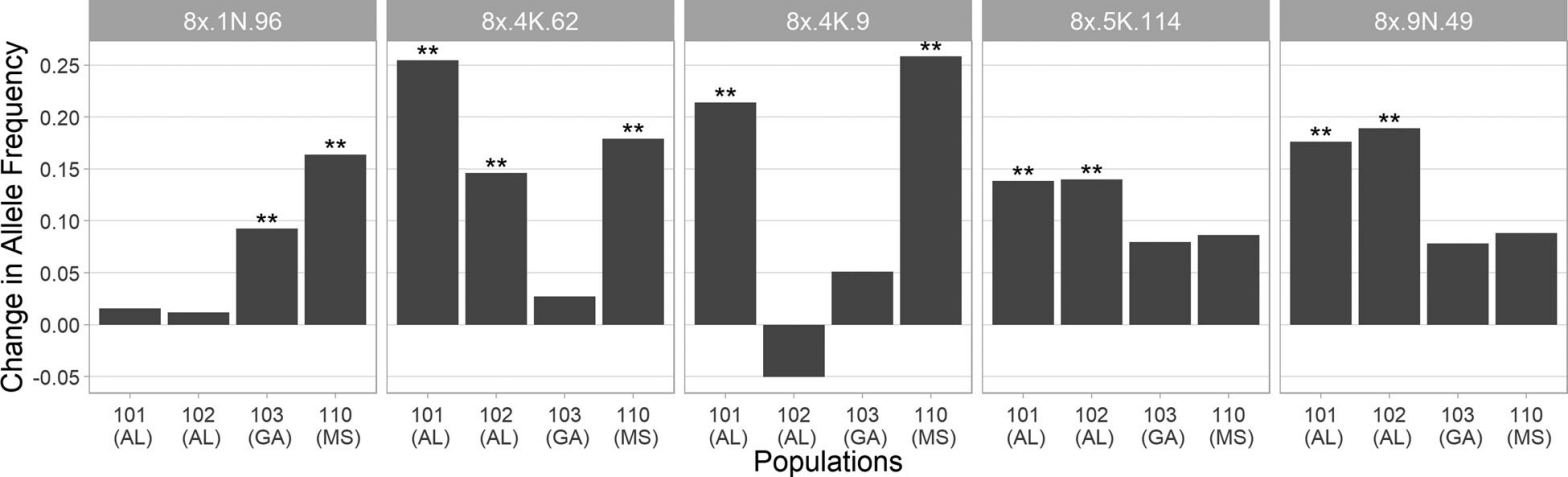
Change in allele frequency for three single nucleotide polymorphism (SNP) markers associated with selection for survivorship (difference between survivor and nonsurvivor groups) for four lowland switchgrass populations evaluated at two Wisconsin locations and classified according to state of origin (** indicates a significant selection effect at *p* < .01)

For the octoploid populations, nine significant TAV comprising seven unique QTL were identified (Table 2). The post‐hoc analysis of these TAV indicated Δ was different (*p* < .01) in at least two out of four populations for six TAV, with the greatest Δ of 0.16 observed for QTL 8x.4K.62. The population from Georgia (WALS_103) contained only one QTL, but this region was associated with differential mortality in both test locations. As in tetraploids, there was no evidence of a selection signature for winter survivorship, as indicated by very small differences in allele frequency between the survival and nonsurvival pool (Figure 5, Table 2, Supplemental Table S1).

A single common QTL region at chromosome 5K has been identified from both tetraploid (115 MB) and octoploid (114 MB) population models. This particular QTL has been found to be associated with seven out of 15 tetraploid populations. The exact SNP of this QTL identified from tetraploid populations (Chr05K_115382984) has not been annotated. This QTL could be considered a promising QTL for improving both tetraploid and octoploid populations simultaneously.

## 4 DISCUSSION

Since the introduction of BSA by Michelmore et al. (1991), it has been successfully implemented in several crops for both qualitative and quantitative traits using dominant or codominant markers, mostly in mapping populations. Examples include cold stress resistance in maize (*Zea mays* L.) (Farooqi et al., 2016), drought resistance in maize (Quarrie et al., 1999), drought resistance in rice (*Oryza sativa* L.) (Salunkhe et al., 2011; Vikram et al., 2012), salt tolerance in rice (Tiwari et al., 2016), Fusarium head blight in wheat (*Triticum aestivum* L.) (Shen et al., 2003), xylose utilization from *Saccharomyces cerevisiae* (Wenger et al., 2010), scab resistance in apple (*Malus domestica* Borkh.) (Yang et al., 1997), and cold tolerance in rice (Sun et al., 2018; Takagi et al., 2013). All these studies used biparental segregating populations for detecting associations. Among the few studies using a diversity panel for quantitative trait association in BSA was a study of kernel numbers in maize (Yang et al., 2015). Bulk segregant analysis had also been successfully used in diverse populations pertaining to the differentiation of adaptive and reproductive mechanisms among ecotypes within species (Gould et al., 2017; Jones et al., 2012; Twyford & Friedman, 2015).

In this study, we aimed to find winter survivorship QTL in natural and BP of lowland switchgrass. These types of populations had undergone numerous historical recombination events and thus had a different genetic structure than segregating populations (Falconer & Mackay, 1996). The power to detect a QTL in natural and BP using BSA is limited mainly due to rapid linkage disequilibrium (LD) decay, accompanied by higher segregation distortion around the QTL as a result of random mating for several generations compared with mapping populations (Quarrie et al., 1999). To account for these spurious associations, smoothing of the G values using kernels is recommended (Magwene et al., 2011). In a biparental or multiparent mapping population, the LD decay rate is slower so the window width of 15–30 cM (Magwene et al., 2011) could be used to smoothen the G values. However, in diverse populations, averaging of the read count by constructing a sliding window with bin size equivalent to LD decay rate is commonly practiced (Gould et al., 2017). In this study, the smoothing of the G‐value with a 250 Kb window size was done based on the results from Ramstein et al. (2016) and Poudel et al. (2019a) who suggested that LD decay is less than 5 cM in a similar diverse panel of lowland switchgrass populations.

The power of the BSA test can be increased by two mechanisms: (a) increasing the number of individuals in the DNA pools and (b) using sufficient sequencing depth (Magwene et al., 2011). In whole‐genome sequencing, a larger pool size increases the accuracy of ‘Pool‐seq’, and, in most comparisons, a pool size of 50 individuals produces sufficiently accurate allele frequency estimates (Schlötterer et al., 2014). Magwene et al. (2011) recommended to use at least 50 individuals per pool from a diversity panel, but in practice BSA analyses were successful with DNA pools of consisting 10–20 individuals in a segregating population from a biparental mating with recombinant inbred lines (Quarrie et al., 1999; Sun et al., 2018). In this study, we bulked only 10% of the individuals (*n* = 8–20) for the survivor and nonsurvivor pools. The detection of significant QTL regions could be affected by Type I error (Navabi et al., 2009), but this error is expected to decrease because of high marker density (Sun et al., 2010). The average read depth on the selected marker used for analysis in this study was only 60. However, this sequencing depth is sufficiently large enough to detect even a weak QTL effect given the bulk size of 20 or more DNA samples (Magwene et al., 2011; Yang et al., 2015).

The analysis was performed separately on tetraploid and octoploid population and accounted for population and evaluation location effects within the logistic regression model with the goal of isolating QTL that will be of practical value for breeding. Despite these controls, we reported different QTL compared with the prior freezing tolerance study on lowland × upland hybrid biparental populations (Poudel et al., 2019b). In addition to the different populations used in these two studies, the discrepancy in the detected QTL could be attributed to the differences in the type of environment used to screen the genotype. Plants react differently to the varying light spectrum, wind, and available soil resources under controlled environment conditions compared with field conditions, and it is not unusual to detect different QTL in different environments (Dhanaraj et al., 2007). Overall, the QTL from a biparental population would be of interest to the lowland–upland hybrids. However, the QTL identified in the current study can be useful to improve multiple populations simultaneously.

### 4.1 QTL across ploidy levels

Octoploids have tetrasomic inheritance and accurate dosage call for intermediate heterozygous classes in octoploids (simplex, duplex, or triplex) require genotyping at high resolution (Ramstein et al., 2018; Uitdewilligen et al., 2013). This highlights an advantage of BSA over the GWAS studies for associating the marker effect in octoploids because BSA relies on sequencing depth for analysis which eliminates the need to classify intermediate heterozygous classes. However, the effects of ploidy on marker effect estimates will need to be considered if the individuals from different ploidy levels are bulked together. In such cases, genome doubling and dominance in intermediate heterozygous classes will impact QTL detection (Endelman et al., 2018) and might affect the ability to detect QTL. In this study, we assessed the QTL from the tetraploid and octoploid populations separately. The QTL regions associated with winter survivorship did not overlap among tetraploid and octoploid populations, with the notable exception of one QTL on chromosome 5K around 114–115 Mb. These results attest to the wide genetic divergence between tetraploid and octoploid lowland switchgrass populations at least in terms of winter survival.

### 4.2 Biological significance of promising QTL

The TAV at QTL 4x.2N.88 codes for *NUCLEOSIDE DIPHOSPHATE KINASE* (*NDPK*) (Phytozome 13.1/*P. virgatum* 4.1). *NDPK* is a stress responsive protein that enhances tolerance to multiple stresses including cold (Kim et al., 2009; Lang et al., 2005; Moon et al., 2003; Sahi et al., 2006). In tall fescue (*Festuca arundinacea* Schreb.), the expression of *NDPK* in plants treated with 200‐mM sodium chloride increased by up to 37‐fold compared with untreated plants (Shahabzadeh et al., 2019).The QTL at chromosome 5K at position ∼114–115 Mb were identified in both octoploid and tetraploid populations with maximum Δ of 0.31 in Kanlow, OK (Supplemental Table S1). The exact peak SNP from the octoploid populations is located within the coding region for the protein CULLIN (Phytozome 13.1/*P. virgatum* 4.1). CULLIN is an important component of the ubiquitin proteasome pathway, which in conjugation with RING E3 ligase degrades ICE1 and is thus regarded as a negative regulator to cold stress (Choi et al., 2014; Dong et al., 2006). Similarly, probable membrane protein *DUF221* protein is collocated with these QTL at 115 Mb. *DUF221* proteins are a family of osmosensitive calcium‐permeable cation channels that regulate cold, drought, and salt tolerance (Ganie et al., 2017; Hou et al., 2014; Sanders et al., 1999). In plants, cold, drought, and salt stresses all stimulate the accumulation of compatible osmolytes and antioxidants (Hasegawa et al., 2000; Xiong et al., 2002). Freezing damage is caused by osmotic dehydration triggered by extracellular ice formation, which leads to cell lysis (Sakai & Larcher, 2012; Sandve et al., 2011; Zhang et al., 2010). As such, the role of *DUF221* proteins in response to freezing can be anticipated, but specific research of this protein under cold or freezing stress has not been reported.

Four out of nine QTL identified in tetraploid populations located on chromosome 9N at positions 1, 9, 26, and 100 Mb. An additional QTL identified from the octoploid populations is also located on chromosome 9N at position 41 Mb. The N subgenome is believed to have received genetic introgression of adaptive loci for key biomass‐related traits from upland to lowland populations (Lovell et al., 2021). Interestingly, *phytochrome B* (*PHYB*) genes are located at positions 9 and 98 Mb (phytozome 13.1/*P. virgatum* 4.1; Bahri et al., 2018), suggesting the relevance of our identified QTL as an adaptation signature to winter survival within the lowland tetraploid switchgrass population. Previous work by Bahri et al. (2018) reported *PHYB*, a gene involved in photoperiod response, was responsible for ecotypic differentiation and population adaption. *PHYB* has been reported to negatively impact cold and/or freezing tolerance in several crops (Franklin & Whitelam, 2007; He et al., 2016; Jiang et al., 2020; Lee & Thomashow, 2012; Wang et al., 2016). The transcript within the QTL 4x.9N.9 encodes for histone *H2A*. Histone modification through acylation is considered to be involved in several stress responses (Kim et al., 2015) and *H2A* has been suggested to play an important role in thermal stress response (Boden et al., 2013; Kumar & Wigge, 2010). Similarly, the SNP peak located in QTL 4x.9N.100 has been described as *HEAT SHOCK 70 kDa protein 1/8* (Table 2), a regulator of heat stress (Sung et al., 2001) that may have an important role as plants recover from freezing injury. A low temperature and salt responsive protein *LTI6* (Pavir.9NG688000) is co‐located with this QTL (Phytozome 13.1/*P. virgatum* 4.1). The candidate genes and proteins co‐located with our identified QTL did not show strong selection signature for winter survivorship across multiple lowland populations (data not shown), which could partly be due to the neutralizing allelic effect as a result of bulking populations with different genetic background. These suggestive QTL need further validation for use in marker‐based selection schemes but can possibly be incorporated into genomic selection protocols to improve selection accuracy.

### 4.3 Future prognosis

Overall, this study has established a better understanding of the genetic architecture of winter survivorship in lowland switchgrass and we provide a starting point for marker‐based selection towards improving winter survival in lowland switchgrass. We did not identify any conserved regions for winter survivorship among the populations and thus infer that winter survival in switchgrass is regulated by many QTL with small additive effects which may often be population specific. We propose that the QTL detected in populations across the broad region of focus in this study, including the Gulf Coast and Southern Great Plains, can be used to improve multiple populations simultaneously. Conversely, QTL detected only in specific populations will be limited to the improvement of those populations only. Understanding and exploiting these QTL should focus on the development of lowland switchgrass for northern latitudes with reduced winter mortality. These results suggest that it should be possible to develop additional and broader‐based lowland switchgrass populations adapted to USDA hardiness zones 4 and 5, using germplasm from hardiness zones 7 through 9.

Simultaneous stacking of the all 17 putative QTL identified in this study would be logistically challenging, except through genome‐wide approaches (Collard et al., 2005). For most marker‐based selection approaches, QTL are prioritized based on their proportion of phenotypic variation (Ribaut & Betrán, 1999), but a distinct disadvantage of BSA is the absence of QTL effect estimates. Therefore, we can instead prioritize significant QTL based on biological annotations, the reliability of associations across multiple populations, and relation between allele frequencies and long‐term minimum temperature of the coldest month of the site of origin of population under study. Based on source of information, four QTL are considered most promising: 4x.2N.88, 4x.9N.1, 4x.5K.115 or 8x.5K.114, and 4x.9N.100. The first two QTL showed significant associations to minimum temperature and reliable changes in allele frequency across multiple populations. The QTL 4x.5K.115 or 8x.5K.114 that encodes for the *CULLIN* protein were simultaneously identified from the same genomic region in both tetraploid and octoploid populations. The QTL 4x.9N.100 is considered promising as it encodes for *HEAT SHOCK 70 kDa protein 1/8*. Designing PCR‐based flanking markers to easily identify these QTL could allow rapid identification of superior genotypes across diverse southern lowland populations for adaptation to the northern United States.

## AUTHOR CONTRIBUTIONS

Hari P. Poudel: hari.poudel@agr.gc.ca Formal analysis, Methodology, Project administration, Writing‐original draftx. Neal W. Tilhou: Formal analysis, Writing‐review & editing. Millicent D. Sanciangco: Data curation, Formal analysis, Writing‐review & editing. Brieanne Vaillancourt: Data curation, Writing‐review & editing. Shawn M. Kaeppler: Writing‐review & editing. C. Robin Buell: Conceptualization, Funding acquisition, Writing‐review & editing. Michael D. Casler: Conceptualization, Funding acquisition, Project administration, Supervision, Writing‐review & editing.

## CONFLICT OF INTEREST

The authors declare that there is no conflict of interest.

## Supporting information

Supplemental Figure S1. The trend of (a) minimum temperature and (b) snow depth during October to March in year 2015/16 and 2016/17 at the two locations: Arlington and Madison, WI. Each dot represents the daily average, and the solid line represents the fitted cubic spline with 95% confidence interval.

Supplemental Figure S2. The distribution of allele count and standard error of the allele count across ploidy level (a) for non‐survival and (b) survival pools. The statistics are based on ∼329 K SNP markers with 15 < read depth < 600 and standard error of mean > 4.

Supplemental Table S1. The allele count in each population corresponding to the significant SNP identified in this study. The data have been used for post‐hoc analysis.

## Data Availability

Raw reads are available in the National Center for Biotechnology Sequence Read Archive under BioProject PRJNA529229. Due to size, the SNP data matrix containing read counts of 80 bulked samples extracted for 5,596,351 bi‐allelic loci is available as WALS_BSA_ReadCount_SNP_Matrix.txt.gz on the Dryad Digital Repository at https://doi.org/10.5061/dryad.9cnp5hqhv.
